# Correspondence

**Published:** 1860-12

**Authors:** Christopher Goodbrake


### CORRESPONDENCE—DIPHTHERIA.

Prof. N. S. Davis:

Diphtheria is just at this time very prevalent in De Witt County, and in some portions of the county it has proved very fatal, especially in the neighborhoods of Marion and Mt. Pleasant. We have also had several deaths from this disease in Clinton and vicinity.

The best treatment, so far as my knowledge extends, is the internal administration of chlorate of potassa, quinine, mur. tinct. iron ; and I have frequently given the syr. of the iodide of iron with good effect. As a local application to the fauces I give the preference to the tr. ferri. mur. in its full strength ; and occasional gargles of brandy, as also of an infusion of capsicum and common salt—a tablespoonfull of each to half a pint of hot water, to this when strained, is added half a pint of hot vinegar.

Owing to the great tendency to prostration in this disease, I have never given emetics of tart. ant. ct. pot., neither have I given calomel, either in alterative doses or as a purge. On the contrary, I pursue the supporting treatment altogether. In the tonic treatment above mentioned I direct my patients to have freely of beef broth, as also brandy or good porter, according to the virulence of the disease, and the age of the patient. When necessary to move the bowels, I prefer mild doses of castor oil, or the seidslitz mixture. With this treatment

I have had tolerable good success, having lost but few patients in proportion to the number treated.

I should be pleased to read an article on the treatment of the disease, under consideration, from the senior editor of the *Examiner.*

Very truly, yours,

CHRISTOPHER GOODBRAKE.

As everything in relation to the treatment of this very troublesome disease is of interest to our readers, at the present time, we copy the following from the proceedings of a recent meeting of the New York Medico Chirurgical College, as reported in the *World.*—( EDITOR OF EXAMINER.)

On thursday evening a meeting of this Medical Society was held, at which Dr. Dewees presided. A very curious specimen of diphtheritic membrane was exhibited by Dr. Sayre, which was expelled from the throat of one of his infant patients about four hours previous to the meeting. The child had been kept four or five days in a room filled with the vapor of water, and heated permanently to the temperature of 85° F. By this means, the membrane, which would otherwise have hardened, and inevitably have suffocated the patient, was kept soft, and any new membraneous material formed in the air passages was dissolved, and thrown off by expectoration. At length, the disease having run its course, the layer of this material which had been first deposited within the windpipe, was loosened and got rid of by the violent explosive coughing of the patient. The membrane exhibited formed a beautiful and perfect cast of the interior of the trachea, and the patient having been delivered from this foreign substance, was out of danger. Dr. Sayre mentioned several cases in which he had been similarly successful by this mode of treatment, which consisted of two things: First, the atmosphere of the room was kept saturated, loaded with moisture, as this was the best, perhaps the only efficient means of dissolving the diphtheritic membrane and preventing it closing the air-tubes. Secondly, the strength of the patient was sustained until the violence of the disease had been spent. For this purpose, brandy and other suitable stimulants were given.

